# Mesoscale Modeling of Hydrogels Under Frictional Shear Stress

**DOI:** 10.1021/acs.macromol.5c01748

**Published:** 2025-11-06

**Authors:** Mehdi Karimi, Amir Poorghani, Angela A. Pitenis, Alexander Alexeev

**Affiliations:** † George W. Woodruff School of Mechanical Engineering, 1372 Georgia Institute of Technology, Atlanta, Georgia 30332, United States; ‡ Materials Department, University of California, Santa Barbara, Santa Barbara, California 93106, United States

## Abstract

Hydrogels are three-dimensional networks of hydrophilic polymers often used as a simplified model of hydrated biological materials, from cartilaginous joints to the ocular tear film. However, the lubrication mechanisms of hydrogels remain poorly understood, partly due to their complex polymeric structure, which creates blurred interfaces during sliding that are challenging to study experimentally. In this study, we employ dissipative particle dynamics (DPD) to investigate the frictional behavior of a polymeric hydrogel network sliding against a solid wall in an explicit viscous solvent. This computational approach enables us to model hydrodynamic interactions and mesoscale polymer dynamics, capturing key aspects of hydrogel friction. Our simulations reveal that hydrogel friction is governed by the interplay between polymer relaxation and viscous shear, characterized by the Weissenberg number (*Wi*). At low *Wi*, friction coefficient remain nearly constant, dominated by polymer relaxation. However, at higher *Wi*, friction is dominated by viscous drag within a near-wall solvent layer, leading to a linear increase in friction coefficient with *Wi*. Furthermore, our results demonstrate an inverse relationship between the friction coefficient and the applied normal load, consistent with experimental observations. This work provides new insights into the fundamental tribological properties of hydrogels, shedding light on the micromechanics of hydrogel friction. Improving our understanding of hydrogel structure and dynamics under friction advances our knowledge of the mechanisms regulating biological lubrication in health and disease.

## Graphical abstract

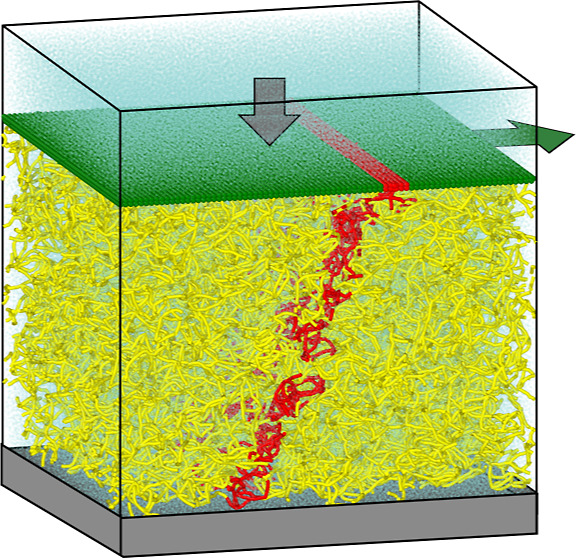

## Introduction

Hydrogels are three-dimensional networks of hydrophilic polymers that swell in water or aqueous solvents.^1–3^ Biological hydrogel networks are responsible for providing exceptionally low friction across natural sliding interfaces such as in articular cartilage and ocular surface.^4^ In some cases, friction coefficients as low as 10^–4^ have been reported.^5,6^ The unique tribological properties make hydrogels promising materials for biomedical and engineering applications where minimal interfacial resistance is essential.^7–9^ In artificial cartilages, ultralow friction is a critical parameter for reducing wear and maintaining smooth joint motion under load.^10–12^ In soft contact lenses, enabling low friction across the cornea–lens interface is important for maintaining comfort and ocular health.^13^ In soft robotics, the use of low-friction hydrogels minimizes abrasion while conforming to delicate surfaces.^14^ Advancing our understanding of hydrogel friction is therefore highly important for both fundamental science and practical engineering applications.^15,16^

Several models have been proposed to explain the mechanisms underlying hydrogel lubrication.^12^ Hydration lubrication and hydrodynamic lubrication are commonly accepted as dominant contributors to low-friction in hydrogels. The hydration lubrication model involves water molecules associated with hydrophilic polymer chains forming a lubricating layer that reduces friction between surfaces, resulting in fluid-like responses to shear forces.^17–19^ Studies have demonstrated that this hydration layer plays a crucial role in minimizing frictional forces by acting as a boundary lubricant, preventing direct contact between sliding surfaces.^20,21^ In contrast, the hydrodynamic lubrication model suggests that at low sliding velocities, friction is primarily controlled by fluid flow through the hydrogel mesh and a mesoscopic liquid film develops between the hydrogel and the solid surface, facilitating lubrication.^20,22^

Experimental studies highlight the role of shear-induced polymer dynamics in controlling hydrogel friction. In experiments with like-charged hydrogels, Oogaki et al.^23^ reported three distinct lubrication regimes, including boundary, hydrated, and elastohydrodynamic regimes, pointing to the existence of multiple frictional mechanisms depending on sliding conditions. Cuccia et al.^20^ demonstrated that hydrogel friction on smooth surfaces exhibits a complex, velocity-dependent behavior comprising multiple regimes, with a notable linear increase in friction coefficient at low velocities attributable to Darcy-like flow through the polymer network and interfacial chain interactions. This regime transitions to a lubricated state at higher velocities, influenced by network mesh size and structural relaxation. Pitenis and Sawyer^24^ examined the lubricity of self-mated hydrogels, including polyacrylamide (PAAm), polyethylene glycol (PEG), and poly­(*N*-isopropylacrylamide) (PNIPAm), and reported a transition from velocity-independent to velocity-dependent friction. This crossover was attributed to a shift from thermally dominated behavior at low sliding velocities to shear-driven deformation and dissipation at higher velocities. Their findings pinpoint the critical role of the Weissenberg number *Wi* in governing the onset of nonlinear frictional behavior in hydrogels. Pitenis et al.^25^ reported that the balance between elastic and viscous responses under shear affects energy dissipation and, consequently, friction forces.

Rennie et al.^26^ investigated the frictional response of hydrogel-based contact lenses and identified three contributing mechanisms to the total friction force: viscoelastic deformation of the hydrogel network, interfacial shear stress due to polymer–surface interactions, and viscous shearing of a thin fluid layer. Their model predicted that the friction coefficient should decrease with load, with an approximate scaling of μ ∼ *P* ^–0.4^ to μ ∼ *P* ^–0.5^, depending on the dominant dissipation mode. Their work provided early experimental evidence for load-dependent friction scaling in soft, hydrated materials. Similarly, Urueña et al.^27^ investigated friction in Gemini hydrogel interfaces and reported a scaling of μ ∼ *P* ^–1/3^ in the velocity-independent regime. They attributed this behavior to a combination of Hertzian contact mechanics, where real contact area increases with load, and constant interfacial shear stress. In their interpretation, friction arises primarily from shearing within a thin, solvent-rich interfacial layer rather than from the bulk gel, and the sublinear dependence reflects the mismatch between the growth of contact area and the frictional force. Shoaib et al.^28^ and Shoaib and Espinosa-Marzal^29^ used lateral force microscopy to investigate the frictional response of hydrogels under varying loads and sliding speeds including static friction. They identified friction regimes dominated by polymer adsorption–desorption, poroelastic relaxation, and network deformation, and reported a sublinear increase in friction force with load. Blum and Ovaert^30^ combined tribological testing with poro-viscoelastic finite element modeling to investigate the frictional behavior of PVA hydrogels functionalized with a DOPA-based boundary lubricant. They demonstrated that surface functionalization significantly reduces friction, and reported a decrease in friction with increasing normal load.

Experimental studies are limited in resolving the nanoscale interactions between the hydrogel network and solvent underpinning the fundamental mechanisms of frictional energy dissipation. Researchers have employed computational modeling to gain insight into hydrogel friction at molecular and mesoscopic scales. Müser et al.^31^ developed a computational model to simulate the frictional response of hydrogels with different stiffness, surface roughness, and confinement, reveling distinct lubrication regimes influenced by gel mechanics. Wu et al.^32^ used molecular dynamics to examine how water content affects the tribological properties of biologically relevant hyaluronic acid hydrogels. Their results show that increasing gel hydration reduces the friction by enhancing molecular mobility and interfacial lubrication. Mees et al.^33^ employed a mesoscale method with implicit solvent to reveal that friction at hydrogel–hydrogel interfaces arises from the entropic stress generated by the reorientation and stretching of surface polymer chains. In particular, they showed that chain reorientation dominates friction at intermediate Weissenberg numbers, yielding a velocity-independent regime for low *Wi* followed by a linearly velocity-dependent regime for the friction coefficient. Similar chain reorientation with *Wi* was found in grafted polymer chains in a shear flow, where chain align with the direction of shear and stretch, resulting in a linear increase of entropic shear stress.^34^ Zhu et al.^35^ used experiments, theoretical modeling, and coarse-grained molecular dynamics (CGMD) simulations with an explicit solvent to probe gel friction in an open-air environment. They showed that hydrogel friction is governed by hydrodynamic drag through the polymeric network and that the friction coefficient is influenced by the hydrodynamic layer thickness, mesh size, and effective viscosity, all of which depend on water transport and polymer–water interactions.

In this work, we develop a model of a hydrogel sliding along a flat solid wall in an explicit solvent to probe its friction. We focus on swollen, chemically cross-linked hydrogels that exhibit a near-surface polymer-depleted layer with a characteristic thickness of roughly one to a few mesh sizes arising from entropic (confinement) and excluded-volume effects. This gel structure is consistent with experiments that demonstrate sparser networks with dangling chains at the interface compared to the bulk.^36–38^ Our model is based on dissipative particle dynamics (DPD), a mesoscale simulation technique that effectively captures both hydrodynamic interactions and polymer dynamics in hydrated polymer networks.^39,40^ We have previously used DPD to model the mechanics of polymeric networks and microgels,^41–43^ demonstrating its capability for probing solvent-mediated mechanics and deformation in hydrogels. Our computational model enables a direct examination of the micromechanics of hydrogel friction by capturing the interactions among a sliding polymeric network, viscous solvent, and a solid substrate. We quantify the effects of sliding velocity and normal load on the friction coefficient. By evaluating local density, deformation, and velocity profiles, we reveal the role of the gel structure and near-wall gel changes in mediating friction forces under load. Our results identify a crossover in frictional behavior governed by the Weissenberg number and demonstrate that viscous drag within the interfacial solvent layer is the dominant source of friction at the higher Weissenberg number regimes.

## Methods

We use dissipative particle dynamics (DPD) to model friction between a solid wall and a polymeric network immersed in an explicit viscous solvent. DPD is a particle-based mesoscale method where particles, representing clusters of molecules, interact via soft potentials. This approach enables simulations to span longer time and larger length scales, making it computationally efficient for studying large dynamic systems over extended time periods. Additionally, DPD employs pairwise interactions that inherently conserve momentum, allowing it to properly capture hydrodynamic interactions critical for investigating hydrogel friction.^44^

In DPD, the total force acting on a DPD particle is given by $F_{i}=\sum_{j\neq i}\left(F_{ij}^{C}+F_{ij}^{D}+F_{ij}^{R}\right)$ , where the force acting on a bead *i* arises from interactions with its neighboring beads *j* within a cutoff radius *r*_c_. The conservative force $F_{ij}^{C}=a_{ij}w\left(r_{ij}\right){\mathrm{r̂}}_{ij}$ represents the repulsion between particles accounting for excluded volume. Here, *a*_*ij*_ is the repulsion parameter, $w\left(r_{ij}\right)=1-\frac{r_{ij}}{r_{c}}$ is the weight function, and ${\mathrm{r̂}}_{ij}$ is the unit vector connecting the particles. The dissipative force $F_{ij}^{D}=-\delta w^{2}\left(r_{ij}\right)\left({\mathrm{r̂}}_{ij}\cdot v_{ij}\right){\mathrm{r̂}}_{ij}$ represents the effects of viscosity, with *δ* being the dissipative coefficient and *v*_*ij*_ the relative velocity between particles. The random force $F_{ij}^{R}=\sigma w\left(r_{ij}\right)\zeta_{ij}{\left(\Delta t\right)}^{-1/2}{\mathrm{r̂}}_{ij}$ accounts for thermal fluctuations, where σ is the noise amplitude, ζ_*ij*_ is a random variable with zero mean and unit variance, and Δ*t* is the time step. The fluctuation–dissipation theorem relates σ and *δ* as σ^2^ = 2*δk*_B_ *T*, where *k*_B_ is the Boltzmann constant and *T* is temperature.

The initial gel network is constructed by randomly distributing cross-link points within a simulation box. Then, neighboring cross-link points are connected by up to four polymer chains.^41,42^ This statistically isotropic protocol yields a permanently cross-linked random polymer network with a thin, sparser outer gel layer on the order of the mesh size, in line with depletion expected for swollen hydrogels.^36–38^ Neighboring polymeric chain beads are connected with a harmonic bond potential $U_{bond}=k_{bond}{\left(r-r_{eq}\right)}^{2}$ , where *k*_bond_ is the bond stiffness, *r*_eq_ is the equilibrium bond length, and *r* is the distance between bonded beads. An angle potential $U_{bend}=k_{bend}\left(1+\cos\theta \right)$ is used to set polymer bending stiffness, where *k*_bend_ is the bending stiffness, and θ is the angle between three consecutive beads.

Figure 1a shows the schematic of the computational model. The bottom surface of the simulation box serves as the stationary solid wall against which the hydrogel slides. The top of the hydrogel network is firmly attached to a moving rigid wall that moves with a constant velocity *V* in the positive *x*-direction. This wall is permeable to the solvent but impermeable to gel. A constant compressive force *F*_n_ is applied to this top wall, which is allowed to move vertically. As a result, the gel network is compressed, and solvent is expelled through the permeable wall, maintaining a constant density in the gel–solvent system. Figure 1b shows a representative snapshot of the simulation.

**Figure 1:**
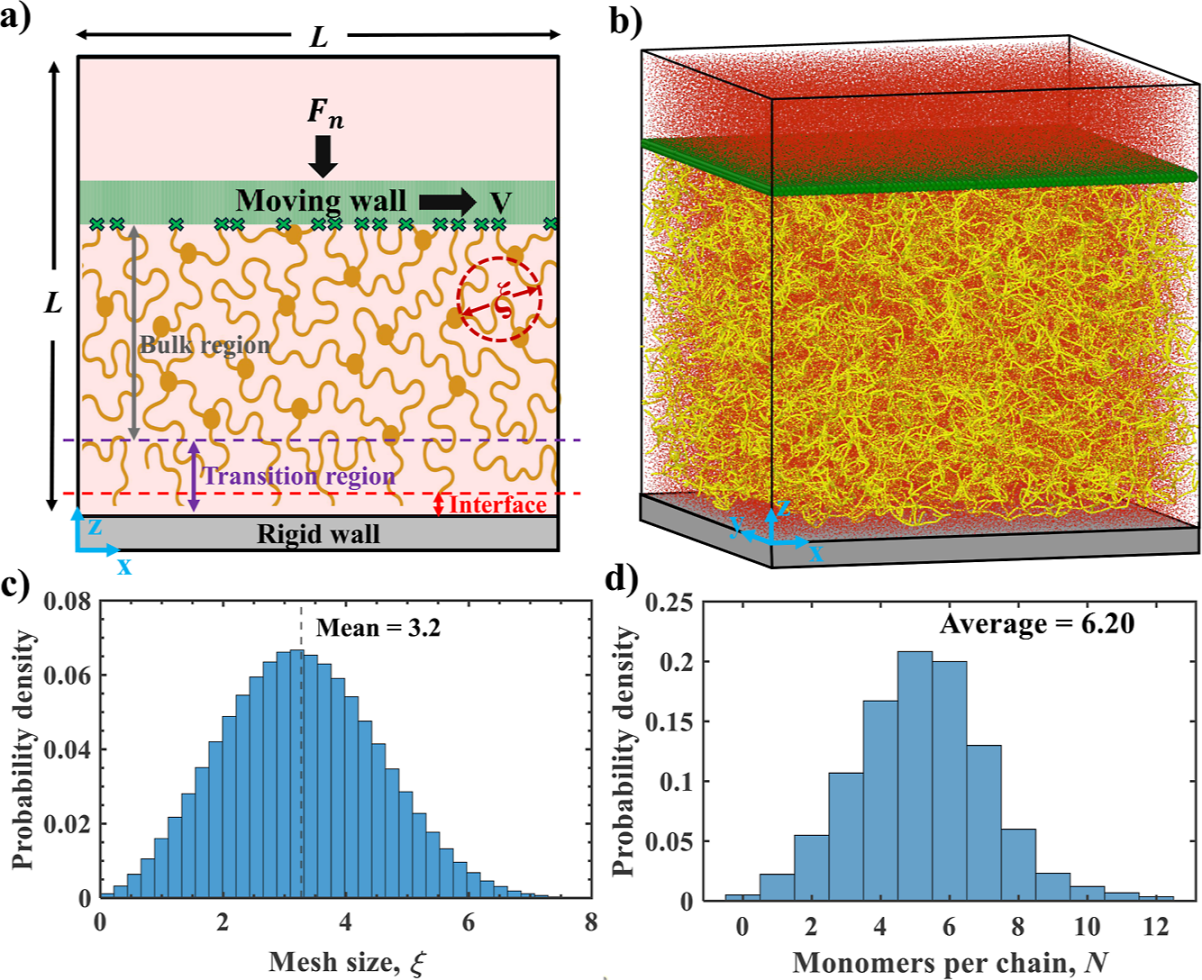
(a) Schematic of the computational setup. A polymeric gel network (yellow) slides along a rigid bottom wall (gray). The gel is attached at the top to a rigid moving wall (green) translating at velocity *V* along *x*-direction. The moving wall is subject to a normal compressive force *F*_n_. The red dashed circle highlights the mesh size ξ of the network. (b) Snapshot of the simulation box showing the three-dimensional gel network (yellow) immersed in an explicit solvent (red), confined between the bottom stationary wall (gray) and the top moving wall (green); periodic boundary conditions are imposed laterally. (c) Probability density distribution of mesh size ξ in the equilibrated gel network. (d) Probability density distribution of contour length *N* (monomers per chain) in the gel network.

Figure 1c quantifies the internal structure of the equilibrated and unloaded gel by presenting the probability density distribution of the network mesh size ξ evaluated with the pore–size algorithm introduced by Gelb and Gubbins.^45^ In this method, the largest solvent-accessible sphere that can be inserted at each point in the polymer network is calculated, yielding a statistical measure of the local mesh size. Figure 1d shows the distribution of the number of monomers *N* in polymer chains connecting cross-linkers within the gel network.

Periodic boundary conditions are imposed on the lateral sides of the simulation box to ensure an infinite repeating hydrogel in the *x*- and *y*-directions. A harmonic repulsion is set at the top box boundary to prevent solvent from leaving the computational domain.

At the bottom boundary that represents the frictional rigid wall, a no-slip boundary condition is applied to the solvent using a bounce-back method.^46^ Specifically, after each integration step, any solvent bead whose center crosses the boundary plane is reflected with its velocity reversed, so both normal and tangential components invert at impact, yielding zero in-plane velocity at the boundary. This boundary condition is supplemented with a harmonic repulsion potential to prevent solvent particles from accumulating next to the bottom box surface. Harmonic potential at the bottom boundary is also used to prevent the gel crossing the rigid wall.

In our simulations, we set *r*_c_ = 1, γ = 4.5, *k*_B_ *T* = 1, Δ*t* = 0.01, the number density of DPD beads ρ_0_ = 3 and the repulsion parameter *a*_*ij*_ = 25. In the hydrogel network model, the mean number of monomers per chain is *N*_0_ = 6.2, the bond stiffness is *k*_bond_ = 16, and the bending stiffness is *k*_bend_ = 10 leading to the hydrogel’s effective elastic modulus *E* = 0.078, porosity ε = 0.89, solvent dynamic viscosity η = 0.84, and a mean mesh size ξ_0_ = 3.2. The porosity ε denotes the solvent-filled volume fraction.

Harmonic potentials of 6.57 and 50 are applied at the bottom wall to the solvent and gel, respectively. At the top boundary, a potential of 50 is applied to the solvent. A domain size of 60 × 60 × 60 is used in the simulations. We verified that our results are independent of the domain size by conducting simulations with a larger domain size. Furthermore, we select the simulation parameters to ensure that the velocities are well below the DPD speed of sound, making compressibility effects negligible, and the maximum shear rate is below 0.1 so that the DPD thermostat keeps temperature variations below one percent.^47^

The simulations were performed for 10^7^ time steps to eliminate the effects of initial transient. The gel data was collected over the last 2 × 10^6^ time steps. The above computational parameters are given in DPD units.

We express the problem in terms of dimensionless groups to facilitate the comparison of our simulation results with other models and experimental data. We introduce a dimensionless normal load *P* = *F*_n_/(*AE*) where *A* is the nominal contact area. The friction coefficient is defined as μ = *F*_f_/*F*_n_, where the friction force *F*_f_ is evaluated as the horizontal force applied to move the hydrogel. To characterize hydrogel sliding, we use the Weissenberg number $Wi=V\eta \xi_{0}^{2}/\left(k_{B}T\right)$ that represents the ratio of polymer relaxation time scale $\eta \xi_{0}^{3}/\left(k_{B}T\right)$ to the time scale of the shear ξ_0_/*V*.^24^ In our simulations, we change *Wi* by changing the sliding velocity *V*. Furthermore, we normalize all distances by the mean mesh size ξ_0_.

We consider *P* in the range between 0.14 and 0.28 and *Wi* in the range between 10^–4^ and 1. These ranges of dimensionless parameters are representative for normal stresses and shear rates reported for ocular^48^ and articular cartilage lubrication.^26,49^ Furthermore, the parameters are relevant to cartilage-mimetic materials with *E* ∼ 0.1–1 MPa^50–52^ and ξ ∼ 50–100 nm.^9,20^

## Results and Discussion

Figure 2a shows the variation of normalized local gel density ρ_g_/ρ_0_ as a function of distance from the rigid wall for different values of Weissenberg number *Wi* and normal load *P*. The distance from the wall is normalized by the characteristic network mesh size ξ_0_. Away from the wall, the gel density remains nearly constant with slight fluctuations with a wavelength on the order of the mesh size, indicating a uniform network structure. We refer to this as the *bulk region*. Closer to the wall, within a distance of approximately 2ξ_0_, the density sharply decreases, reflecting a structural transition at the gel boundary. This drop is associated with a lower cross-link density and the presence of dangling chains near the wall. We refer to this zone of rapid density change as the *transition region*. We further define *interface* as a narrow zone immediately adjacent to the wall at the lower part of the transition region, where the gel network is in direct contact with the rigid wall. The interface thickness is set to 0.3ξ_0_. All three regions are illustrated in Figure 1a.

**Figure 2:**
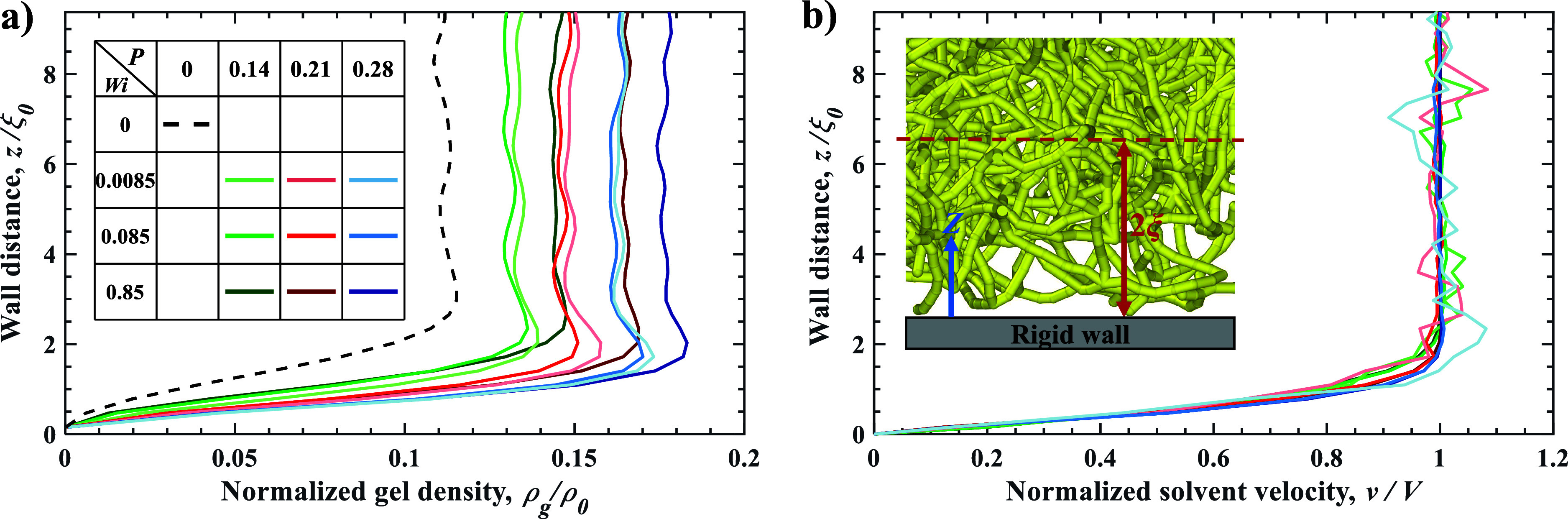
Gel and solvent dynamics. (a) Normalized gel density profile ρ_g_/ρ_0_ versus normalized wall distance *z*/ξ_0_ for different normal loads *P* and Weissenberg numbers *Wi*. The legend in panel (a) indicates the *P* values (by color) and the *Wi* values (by color intensity) and applies to both panels. (b) Normalized solvent velocity profiles *v*/*V* as a function of *z*/ξ_0_ for different *P* and *Wi*. Inset: cropped snapshot of the hydrogel network near the rigid wall.

Changes in *P* have only a modest effect on the thickness of the transition region, resulting in a slight decrease in thickness with increasing *P*. Furthermore, the thickness of the transition region exhibits a slight increase with *Wi*, likely due to a stronger effect of near-wall hydrodynamic lift. Specifically, raising *Wi* from 10^–2^ to 1 enlarges the transition region thickness by approximately 10%. These suggest that an increase in bulk density due to higher *P* and *Wi* leads to a sharper change in ρ_g_, whereas the spatial extent of the structural rearrangement near the wall is relatively insensitive to these parameters.

Figure 2b shows the variation of normalized solvent velocity *v*/*V* with distance from the wall. The solvent velocity is zero at the wall due to the no-slip boundary condition and increases sharply within the transition region, approaching the bulk gel velocity around a distance of 2ξ_0_. This suggests that the spatial extent of the transition region for solvent velocity is similar to that of gel density. Moreover, the normalized solvent velocity profiles for different *P* and *Wi* collapse onto a single curve, indicating that the distribution of solvent velocity near the wall is largely insensitive to *P* and *Wi*. The rapid increase in solvent velocity near the solid wall results from viscous drag between the moving gel filaments and the solvent. Since the gel and solvent move at the same speed in the bulk region, most viscous dissipation is localized in the transition region, playing a critical role in determining gel friction.

The inset in Figure 2b provides a visual representation of the hydrogel network near the rigid wall, highlighting the structural heterogeneity within the transition region. It reveals that the gel exhibits noticeably higher porosity near the wall, which is consistent with the reduced cross-link density and the presence of dangling polymer segments.

Figure 3a presents the normalized bulk gel density ρ_*b*_/ρ_0_ as a function of *Wi* for different values of *P*. For *Wi* < 0.1, corresponding to slower sliding velocities, the bulk gel density is nearly constant and independent of *Wi*. In this regime, the relaxation time of the polymer chains is shorter than the time scale associated with the applied shear deformation. As a result, the polymer chains have sufficient time to equilibrate following mechanical perturbations from sliding. This leads to minor structural changes in the gel network, maintaining a nearly constant bulk gel density at lower *Wi*. For *Wi* > 0.1, ρ_b_ increases with increasing *Wi*. In this regime, polymer network relaxation is slower than the shear time scale, resulting in significant shear deformation of the gel network. This, in turn, leads to a denser packing of the polymer network under compressive load.

**Figure 3:**
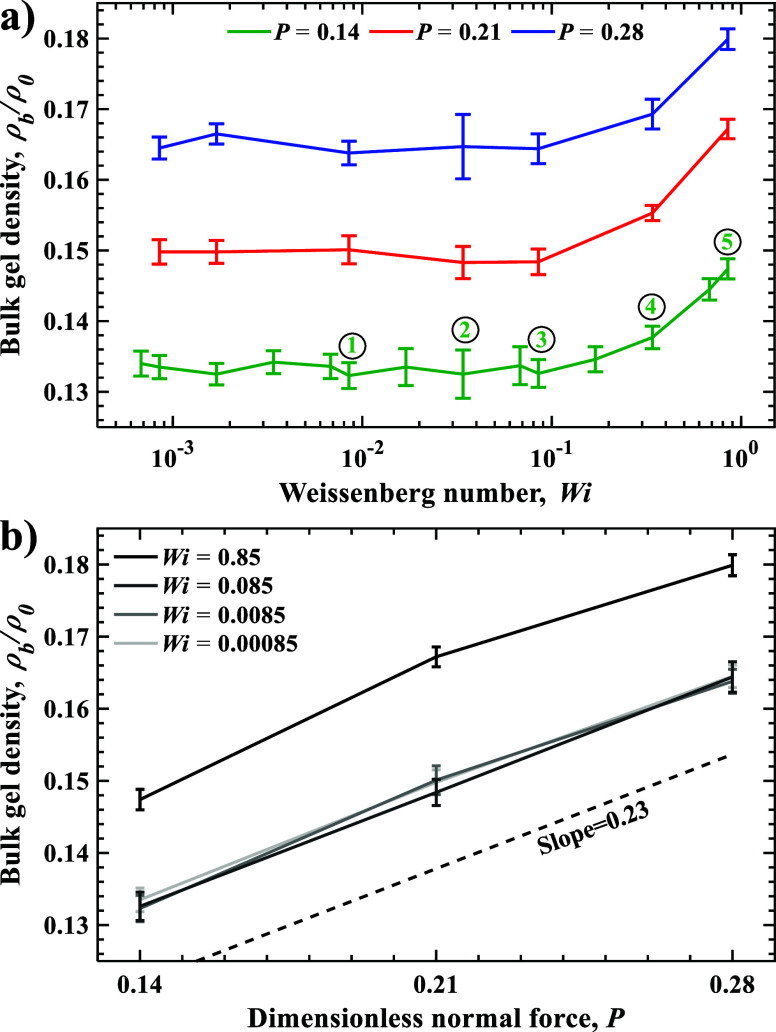
Bulk gel density ρ_b_/ρ_0_ as a function of (a) Weissenberg number *Wi* and (b) normal load *P*. The numbered data points in panel (a) correspond to snapshots in Figure 5. Error bars represent standard deviation.

Figure 3b shows the variation of bulk gel density ρ_b_/ρ_0_ with *P*. We find that ρ_b_ increases nearly linearly with *P*. The slope of this increase is roughly 0.23 and is insensitive to *Wi*. This result suggests that gel compressibility is unaffected by shear-induced structural changes of the polymer network.

Figure 4a,b show the normalized gel density at the interface with the rigid wall ρ_i_/ρ_0_ as a function of *Wi* and *P*, respectively. Figure 4a demonstrates, in contrast to ρ_b_ that increases with *Wi*, ρ_*i*_ slightly decreases with increasing *Wi*. This can be related to an increased contribution of the hydrodynamic lift force displacing the gel away from the rigid wall. This is consistent with Figure 2a, where the thickness of the near-wall transition layer slightly increases with increasing *Wi*, resulting in a broader polymer-depleted zone and reduced interfacial density ρ_i_. Similarly to ρ_b_, the interfacial gel density ρ_i_ increases linearly with *P* (Figure 4b). However, this slope is significantly lower than that for the bulk gel density with Weissenberg number, indicating nearly four-times lower compressibility of the interfacial gel.

**Figure 4:**
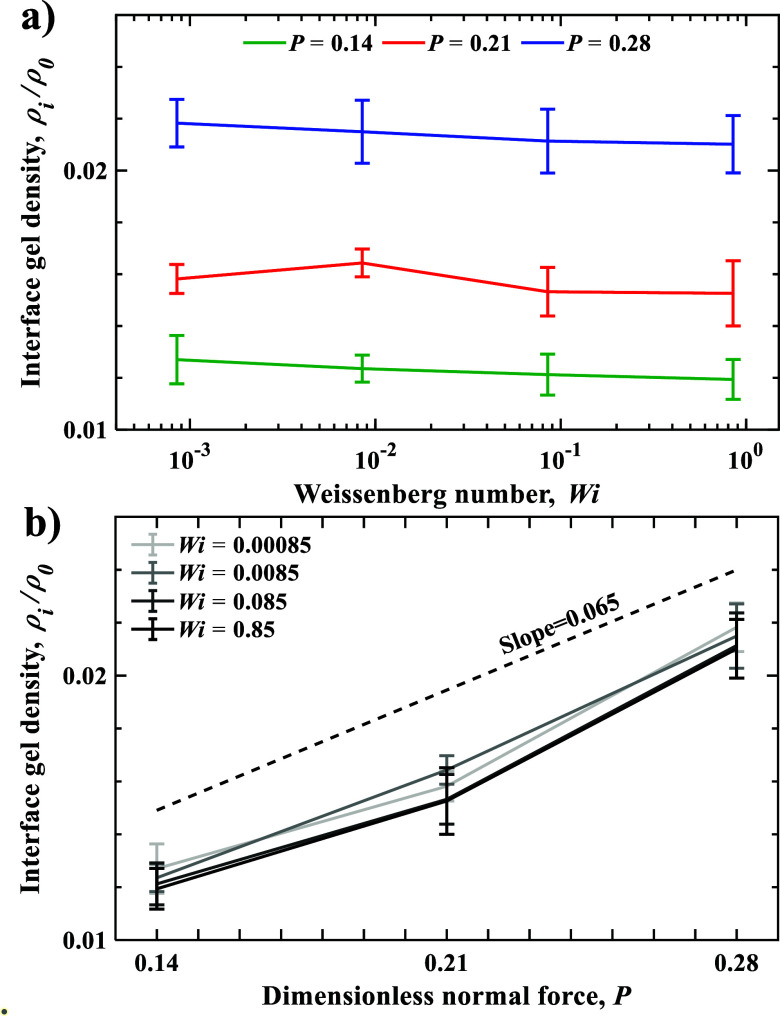
Interfacial gel density ρ_i_/ρ_0_ as a function of (a) Weissenberg number *Wi* and (b) normal load *P*. Error bars represent standard deviation.

Figure 5 quantifies the mean shear strain of the hydrogel γ as a function of *Wi*. Shear strain γ is evaluated as the geometric distortion of the gel with respect to its unloaded configuration. As *Wi* increases, the gel undergoes progressively larger shear deformation, reflected in the increasing γ. For *Wi* < 0.01, γ is nearly zero, indicating that the gel is compressed vertically. For *Wi* > 0.01, γ gradually increases with *Wi*. Note that in this regime, larger *P* leads to a slightly higher γ. The increase in γ with *Wi* is correlated with increased bulk density ρ_*b*_ with *Wi* (Figure 3). This suggests that gel shearing deformation leads to gel compression, which reduces network mesh size.

**Figure 5:**
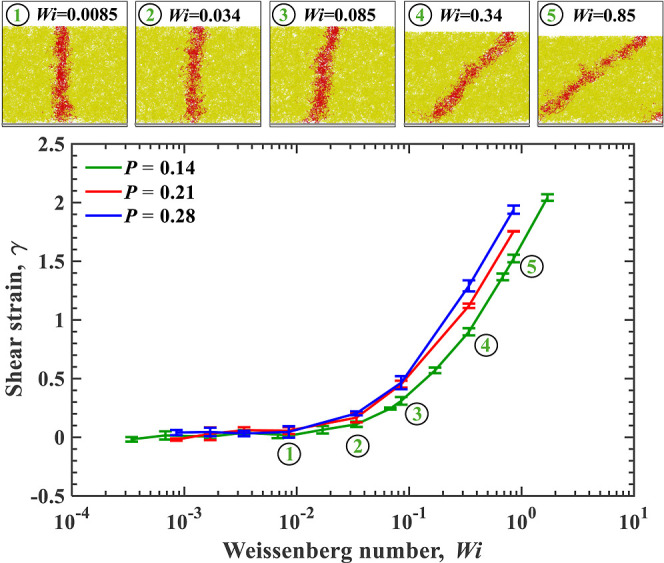
Shear strain γ as a function of the Weissenberg number *Wi* for different normal loads *P*. Snapshots above the plot show the gel deformation for selected *Wi* values labeled 1–5 at *P* = 0.14. Error bars represent standard deviation.

The snapshots in Figure 5 illustrate the steady-state hydrogel network deformation at various *Wi* and *P* = 0.14. The red strip indicates a portion of the gel oriented normally to the sliding surface at *V* = 0. At lower *Wi*, the hydrogel network appears isotropic, with no significant distortion as the red strip remains vertical. As *Wi* increases, the network experiences progressive shear deformation, as indicated by the tilt of the red stripe in the direction of gel motion. The deformation is uniform across the thickness of the gel layer.

Figure 6a presents the variation of normalized friction coefficient μ/μ_0_ as a function of *Wi*. Depending on the magnitude of *Wi*, our simulations reveal two distinct regimes in the friction response of the hydrogel. At low *Wi* < 0.01, the friction coefficient remains nearly constant and independent of *Wi*. For each *P*, we define μ_0_ as the average friction coefficient in the *Wi*-independent regime and use these values to normalize μ. Figure 6a shows that the normalized friction data for different *P* collapse onto a universal curve.

**Figure 6:**
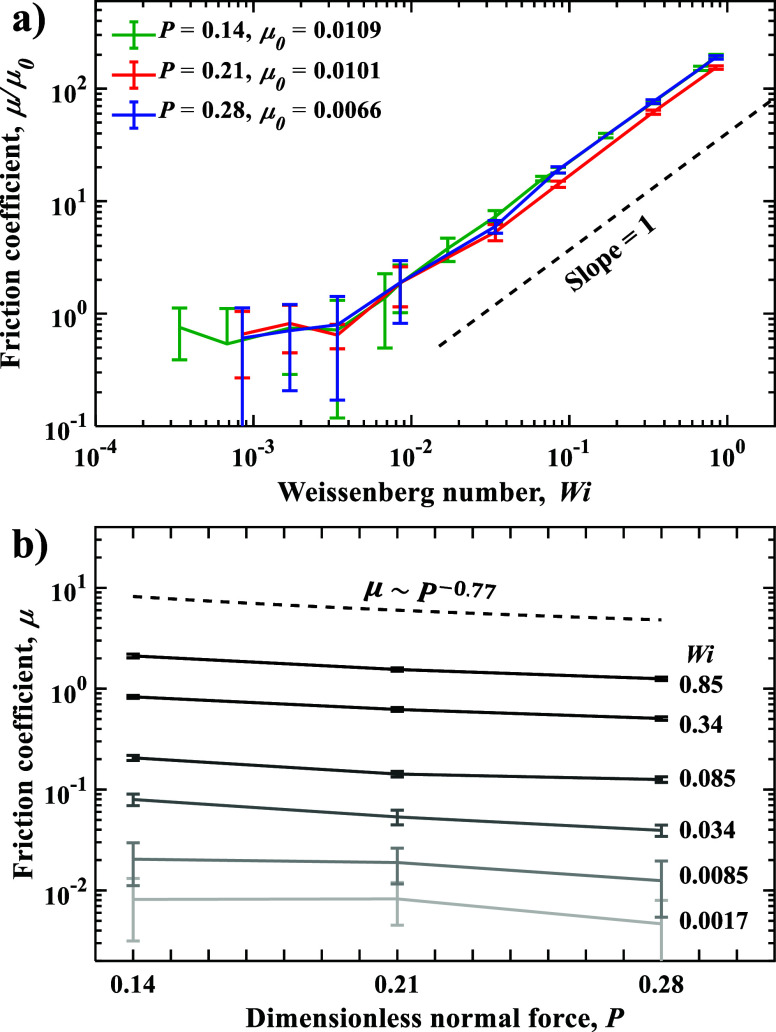
(a) Normalized friction coefficient μ/μ_0_ as a function of Weissenberg number *Wi* for normal loads *P* = 0.14, *P* = 0.21, and *P* = 0.28. (b) Friction coefficient μ as a function of *P* for different values of *Wi*. The dashed line indicates a power-law dependence of −0.77. Error bars represent standard deviation.

The constant gel friction at low *Wi* can be rationalized by the dominance of thermal fluctuations over polymer-chain deformation due to friction. In this regime, the applied shear stress is insufficient to overcome random motion of polymer chains, preventing significant deformation or reorganization of the hydrogel network. As a result, the friction coefficient is independent of *Wi*.

In the high-*Wi* regime, μ increases approximately linearly with *Wi*, suggesting that friction is dominated by solvent hydrodynamics. Recall that in our model the gel does not experience friction interactions with the solid wall. For a constant normal load *F*_*n*_, μ is proportional to the friction force *F*_f_, which in turn is proportional to wall shear stress τ_w_ = η *dv*/*dz*. We assume that the velocity gradient near the wall *dv*/*dz* scales as *V*/*d*, where *d* is the thickness of a thin solvent layer forming between the gel interface and the wall. The solvent-layer thickness *d* scales with the interfacial gel density ρ_*i*_. Indeed, higher *d* requires lower ρ_*i*_. Noting that ρ_*i*_ is nearly independent of *Wi* (Figure 4a) and, therefore, sliding velocity *V*, we conclude that μ scales linearly with *V*, which in turn implies μ ∼ *Wi*, consistent with our simulation.

A similar linear dependence of μ on *Wi* has been observed in coarse-grained MD simulations of self-mated hydrogels^33^ and in experiments with polyacrylamide and agarose hydrogels sliding under nonadhesive contact conditions. In the latter case, the dominant dissipation mechanism was attributed to fluid–network interactions within the hydrogel mesh.^20^ Our simulations show similar frictional scaling, reinforcing the idea that interfacial viscous drag, rather than adhesive or entropic effects, governs friction in this high-*Wi* regime. The difference in the magnitude of friction coefficients between simulation and experiment likely stems from differences in polymer structure, cross-linking density, and solvent–polymer interactions.

Figure 6b shows the variation of the friction coefficient μ as a function of normal load *P*. For all *Wi*, μ decreases monotonically with increasing *P*. The scaling of μ with *P* follows a power-law behavior with an exponent about −0.77, indicating that *F*_*f*_ ∼ *P* ^0.23^. Increasing the normal load densifies the gel, particularly at the interface near the wall (Figure 4b), thereby decreasing the thickness of the sheared solvent layer *d*. This results in increasing *F*_*f*_ with *P*. However, the increase is relatively moderate as ρ_*i*_ increases with *P* significantly slower than the bulk density ρ_*b*_.

Inverse dependence of μ on *P* is consistent with experiments.^26,27^ Although the reported exponents range between −1/2 and −1/3, those differences stem from varying assumptions about contact area growth with load. In our simulations, the nominal contact area is constant, and the observed trend can be attributed to increasing gel density at the interface with the sliding wall. Our results are also consistent with explicit-solvent CGMD simulations,^35^ which likewise found that the friction coefficient decreases with increasing normal load.

## Conclusions

We developed a mesoscale model to investigate the frictional behavior of hydrogels sliding along a rigid flat substrate in an explicit solvent environment. Our simulations capture the essential micromechanical interactions between the hydrogel polymeric network, viscous solvent, and solid substrate, enabling us to probe the interplay between structural deformation and viscous dissipation at mesoscopic scales. In our computational model, a no-slip boundary condition is imposed on the viscous fluid at the solid substrate, whereas the compressed by an external load hydrogel is allowed to slide freely along the substrate. We systematically vary hydrogel load and sliding velocity to reveal the micromechanics of gel friction.

We found that hydrogel friction exhibits two distinct regimes governed by the Weissenberg number *Wi*. At low *Wi*, the friction coefficient μ remains nearly constant, reflecting thermally dominated dynamics with minimal structural rearrangement. In contrast, at higher *Wi*, μ increases linearly with *Wi*, indicating a transition to shear-dominated behavior where viscous drag within a near-wall solvent layer becomes the primary source of friction. Notably, we observed that the thickness of this interfacial solvent layer near the wall, where solvent experiences the maximum velocity gradient, is largely independent of *Wi*, leading to a linear dependence of μ on *Wi* consistent with experimental data.

Our results also demonstrate that increasing normal load *P* leads to densification of the hydrogel, both in the bulk and near the wall, though the compressibility at the interface is significantly lower. This densification decreases the thickness of the sheared solvent layer and contributes to an inverse power-law dependence of μ on *P* with an exponent of approximately −0.77.

These findings highlight the critical role of interfacial solvent dynamics and network deformation in mediating hydrogel friction. By explicitly modeling both the polymer network and solvent, our approach bridges the gap between molecular–scale interactions and continuum-level frictional responses. This framework can be directly applied for designing soft materials with tailored tribological properties. Furthermore, the results facilitate our understanding of the role of gel mechanics in regulating biological lubrication.

## Data Availability

The initial hydrogel geometry used in this study is available at https://github.com/gt-cfms/Mesoscale-Modeling-of-Hydrogels-under-Frictional-Shear-Stress. The repository includes files containing a bead–spring network that models a cross-linked gel structure, formatted for use with LAMMPS.

## References

[1] Ahmed E. M. (2015). Hydrogel:
Preparation, characterization, and applications: A review. J. Adv. Res..

[2] Zhang Y. S., Khademhosseini A. (2017). Advances in
engineering hydrogels. Science.

[3] Ho T.-C., Chang C.-C., Chan H.-P., Chung T.-W., Shu C.-W., Chuang K.-P., Duh T.-H., Yang M.-H., Tyan Y.-C. (2022). Hydrogels:
Properties and Applications in Biomedicine. Molecules.

[4] Gong J. P. (2006). Friction
and lubrication of hydrogels—its richness and complexity. Soft Matter.

[5] Gong J., Iwasaki Y., Osada Y., Kurihara K., Hamai Y. (1999). Friction of
gels. 3. Friction on solid surfaces. J. Phys.
Chem. B.

[6] Murakami T., Yarimitsu S., Nakashima K., Sakai N., Yamaguchi T., Sawae Y., Suzuki A. (2015). Biphasic and boundary lubrication
mechanisms in artificial hydrogel cartilage: A review. Proc. Inst. Mech. Eng., Part H: J. Eng. Med..

[7] Caló E., Khutoryanskiy V. V. (2015). Biomedical applications of hydrogels:
A review of patents
and commercial products. Eur. Polym. J..

[8] Yuk H., Zhang T., Lin S., Parada G. A., Zhao X. (2016). Tough bonding
of hydrogels to diverse non-porous surfaces. Nat. Mater..

[9] Li J., Mooney D. J. (2016). Designing hydrogels
for controlled drug delivery. Nat. Rev. Mater..

[10] Yarimitsu S., Sasaki S., Murakami T., Suzuki A. (2016). Evaluation
of lubrication
properties of hydrogel artificial cartilage materials for joint prosthesis. Biosurf. Biotribol..

[11] Zhang X., Lou Z., Yang X., Chen Q., Chen K., Feng C., Qi J., Luo Y., Zhang D. (2021). Fabrication and characterization
of a multilayer hydrogel as a candidate for artificial cartilage. ACS Appl. Polym. Mater..

[12] Gong J., Osada Y. (1998). Gel friction: A model based on surface repulsion and adsorption. J. Chem. Phys..

[13] Ngai, V. ; Medley, J. ; Jones, L. ; Forrest, J. ; Teiehroeb, J. Tribology and Interface Engineering Series; Elsevier, 2005; Vol. 48, pp 371–379.

[14] Dong X., Wang C., Song H., Shao J., Lan G., Zhang J., Li X., Li M. (2024). Advancement in soft
hydrogel grippers: Comprehensive insights into materials, fabrication
strategies, grasping mechanism, and applications. Biomimetics.

[15] Gong J., Higa M., Iwasaki Y., Katsuyama Y., Osada Y. (1997). Friction of gels. J. Phys. Chem. B.

[16] Xu Z., Lu J., Lu D., Li Y., Lei H., Chen B., Li W., Xue B., Cao Y., Wang W. (2024). Rapidly damping hydrogels
engineered through molecular friction. Nat.
Commun..

[17] Cao Y., Klein J. (2022). Lipids and lipid mixtures
in boundary layers: From hydration lubrication
to osteoarthritis. Curr. Opin. Colloid Interface
Sci..

[18] Bahrami M., Houérou V. L., Rühe J. (2022). Lubrication mechanism of surface-attached
hydrogel layers in sliding contact. Adv. Mater.
Interfaces.

[19] Ma L., Gaisinskaya-Kipnis A., Kampf N., Klein J. (2015). Origins of
hydration
lubrication. Nat. Commun..

[20] Cuccia N.
L., Pothineni S., Wu B., Méndez Harper J., Burton J. C. (2020). Pore-size dependence
and slow relaxation of hydrogel
friction on smooth surfaces. Proc. Natl. Acad.
Sci. U.S.A..

[21] Lin, W. ; Kluzek, M. ; Cao, Y. ; Klein, J. Bioinspired polymer-incorporating self-lubricating and antifouling hydrogels. arXiv preprint arXiv:2404.05234 2024.

[22] Shoaib T., Espinosa-Marzal R. M. (2020). Advances in Understanding Hydrogel
Lubrication. Colloids Interfaces.

[23] Oogaki S., Kagata G., Kurokawa T., Kuroda S., Osada Y., Gong J. P. (2009). Friction between like-charged hydrogels—combined
mechanisms of boundary, hydrated and elastohydrodynamic lubrication. Soft Matter.

[24] Pitenis A. A., Sawyer W. G. (2018). Lubricity of high
water content aqueous gels. Tribol. Lett..

[25] Pitenis A. A., Urueña J. M., Schulze K. D., Nixon R. M., Dunn A. C., Spencer N. D., Sawyer W. G. (2014). Hydrogel mechanics
and friction:
Fundamentals and applications. Soft Matter.

[26] Rennie A., Dickrell P., Sawyer W. (2005). Friction coefficient
of soft contact
lenses: Measurements and modeling. Tribol. Lett..

[27] Urueña J. M., McGhee E. O., Angelini T. E., Dowson D., Sawyer W. G., Pitenis A. A. (2018). Normal load scaling
of friction in gemini hydrogels. Biotribology.

[28] Shoaib T., Heintz J., Lopez-Berganza J. A., Muro-Barrios R., Egner S. A., Espinosa-Marzal R. M. (2018). Stick–slip
friction reveals
hydrogel lubrication mechanisms. Langmuir.

[29] Shoaib T., Espinosa-Marzal R. M. (2019). Influence
of loading conditions and temperature on
static friction and contact aging of hydrogels with modulated microstructures. ACS Appl. Mater. Interfaces.

[30] Blum M. M., Ovaert T. C. (2012). Experimental and numerical tribological studies of
a boundary lubricant functionalized poro-viscoelastic PVA hydrogel
in normal contact and sliding. J. Mech. Behav.
Biomed. Mater..

[31] Müser M. H., Li H., Bennewitz R. (2019). Modeling the
contact mechanics of hydrogels. Lubricants.

[32] Wu Y., Wang F., Shi Y., Lin G., Qiao J., Wang L. (2022). Molecular dynamics simulation of hyaluronic acid hydrogels: Effect
of water content on mechanical and tribological properties. Comput. Methods Programs Biomed..

[33] Mees J., Simič R., O’Connor T. C., Spencer N. D., Pastewka L. (2023). Molecular
mechanisms of self-mated hydrogel friction. Tribol. Lett..

[34] Mees J., O’Connor T. C., Pastewka L. (2023). Entropic stress of grafted polymer
chains in shear flow. J. Chem. Phys..

[35] Zhu W., Li J., Du F., Jian N., Wang J., Zhang K. (2025). Friction behavior
and microscopic mechanism of hydrogels in an open-air environment. Adv. Mater..

[36] Rubinstein, M. ; Colby, R. H. Polymer Physics; Oxford University Press, 2003.

[37] Kiyama R., Yoshida M., Nonoyama T., Sedlačík T., Jinnai H., Kurokawa T., Nakajima T., Gong J. P. (2023). Nanoscale
TEM imaging of hydrogel network architecture. Adv. Mater..

[38] Chau A. L., Edwards C. E., Helgeson M. E., Pitenis A. A. (2023). Designing
superlubricious
hydrogels from spontaneous peroxidation gradients. ACS Appl. Mater. Interfaces.

[39] Irfachsyad D., Tildesley D., Malfreyt P. (2002). Dissipative particle dynamics simulation
of grafted polymer brushes under shear. Phys.
Chem. Chem. Phys..

[40] Soddemann T., Dünweg B., Kremer K. (2003). Dissipative particle dynamics: A
useful thermostat for equilibrium and nonequilibrium molecular dynamics
simulations. Phys. Rev. E.

[41] Masoud H., Alexeev A. (2010). Permeability and diffusion through mechanically deformed
random polymer networks. Macromolecules.

[42] Nikolov S., Fernandez-Nieves A., Alexeev A. (2018). Mesoscale modeling of microgel mechanics
and kinetics through the swelling transition. Appl. Math. Mech..

[43] Nikolov S. V., Fernandez-Nieves A., Alexeev A. (2020). Behavior and mechanics of dense microgel
suspensions. Proc. Natl. Acad. Sci. U.S.A..

[44] Groot R. D., Warren P. B. (1997). Dissipative particle dynamics: Bridging
the gap between
atomistic and mesoscopic simulation. J. Chem.
Phys..

[45] Gelb L. D., Gubbins K. E. (1999). Pore size distributions
in porous glasses: A computer
simulation study. Langmuir.

[46] Revenga M., Zuniga I., Espanol P., Pagonabarraga I. (1998). Boundary models
in DPD. Int. J. Mod. Phys. C.

[47] Boromand A., Jamali S., Maia J. M. (2015). Viscosity measurement
techniques
in dissipative particle dynamics. Comput. Phys.
Commun..

[48] Kapadia W., Qin N., Zhao P., Phan C.-M., Haines L., Jones L., Ren C. L. (2022). Shear-thinning
and temperature-dependent viscosity
relationships of contemporary ocular lubricants. Transl. Vision Sci. Technol..

[49] Wong B. L., Bae W. C., Gratz K. R., Sah R. L. (2008). Shear deformation
kinematics during cartilage articulation: Effect of lubrication, degeneration,
and stress relaxation. Mol. Cell. Biomech..

[50] Gong J. P. (2010). Why are
double network hydrogels so tough?. Soft Matter.

[51] Haque M. A., Kurokawa T., Gong J. P. (2012). Super tough double network hydrogels
and their application as biomaterials. Polymer.

[52] Fuchs S., Shariati K., Ma M. (2020). Specialty
tough hydrogels and their
biomedical applications. Adv. Healthcare Mater..

